# Papules confined to black-ink tattoos

**DOI:** 10.1016/j.jdcr.2025.07.013

**Published:** 2025-08-07

**Authors:** Veronica Voronina, Heidi Bai, Dylan J. Badin, Robert E. LeBlanc, Joi B. Carter, Dorothea T. Barton

**Affiliations:** a Geisel School of Medicine at Dartmouth, Hanover, New Hampshire; b Department of Dermatology, Dartmouth Health Medical Center, Lebanon, New Hampshire; c Department of Pathology & Laboratory Medicine, Dartmouth Health Medical Center, Lebanon, New Hampshire

**Keywords:** cutaneous sarcoidosis, immune checkpoint inhibitor, melanoma, PD-1 inhibitor, pembrolizumab, sarcoid-like granulomatous reaction, sarcoid-like reaction, sarcoidosis, sarcoidosis-like granulomatous reaction, sarcoidosis-like reaction, tattoo

## History

**Fig 1:**
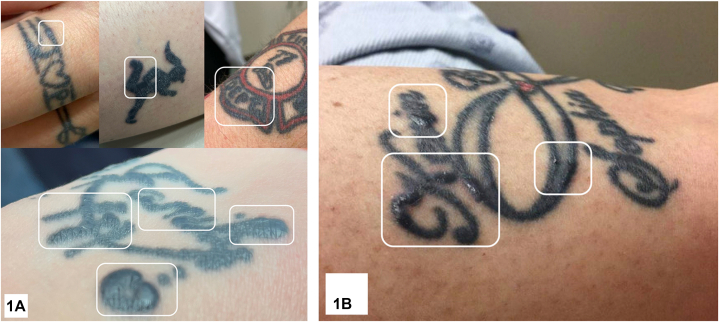

**Fig 2:**
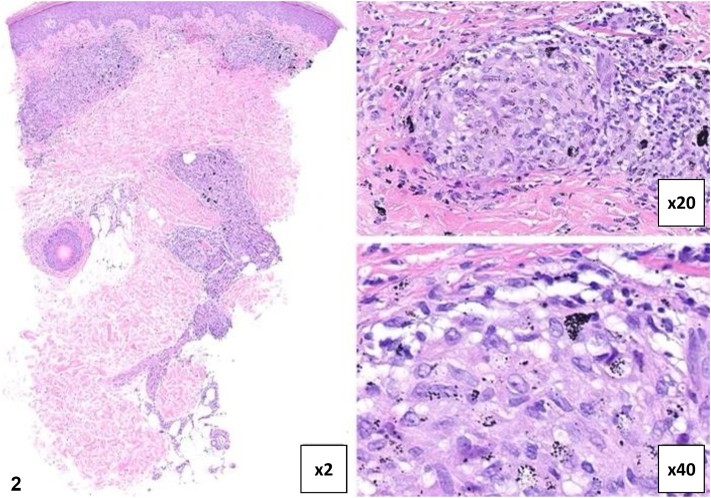

Patient 1 is a 65-year-old female with papules confined to black tattoo ink after 2 months of pembrolizumab for metastatic melanoma (Fig 1, *A*). Punch biopsy is shown (Fig 2). She reported shortness of breath and chest computed tomography revealed ground-glass opacities and intrathoracic lymphadenopathy. Patient 2 is a 45-year-old female who developed erythematous papules within black tattoo ink after 4 months of pembrolizumab for metastatic melanoma (Fig 1, *B*). Chest computed tomography showed paratracheal node enlargement. In both patients, dermatologic and pulmonary findings resolved after discontinuing pembrolizumab and starting prednisone.

**Question 1: What is the most likely diagnosis in both cases?**

A. Tattoo hypersensitivity reaction
B. Sarcoid-like granulomatous reaction
C. Cutaneous sarcoidosis
D. Foreign body granuloma
E. Keloids

**Answers:**

A. Tattoo hypersensitivity reaction – Incorrect. Tattoo hypersensitivity reactions can be heterogenous in presentation; however, the clinical history makes this diagnosis less likely.
B. Sarcoid-like granulomatous reaction – Correct. Sarcoidosis-like reactions (SLRs) secondary to pembrolizumab have been reported, occurring in 1.4% of patients in phase 3 clinical trials.^1^ These reactions can present with noncaseating granulomas in the skin, lungs, and lymph nodes. In both patients, cutaneous findings were localized to black tattoo ink, with no red pigment involvement. The resolution of dermatologic and pulmonary symptoms, in both patients, after pembrolizumab discontinuation and starting corticosteroids supports this diagnosis.
C. Cutaneous sarcoidosis – Incorrect. SLRs differ from true sarcoidosis due to their identifiable pharmacologic trigger.
D. Foreign body granuloma – Incorrect. Localized foreign body reactions to exogenous materials like sutures or tattoo ink would not explain the systemic features observed.
E. Keloids – Incorrect. Keloids demonstrate excess collagen, not granulomatous inflammation.

**Question 2: What is the expected histopathologic finding in the paratracheal lymph node biopsy from patient 2?**

A. Noncaseating granulomas
B. Caseating granulomas
C. Central necrosis with a neutrophilic infiltrate
D. Sheets and aggregates of atypical lymphoid cells
E. Atypical melanocytes with pagetoid spread

**Answers:**

A. Noncaseating granulomas – Correct. Noncaseating granulomas are the hallmark of SLRs. In these patients, histology, systemic symptoms, and cutaneous findings pointed to a diagnosis of immunotherapy-associated granulomatous inflammation. Complete systemic and cutaneous symptom resolution after pembrolizumab discontinuation and systemic corticosteroid treatment initiation further supported this diagnosis.
B. Caseating granulomas – Incorrect. Caseating granulomas are typically found in infections such as tuberculosis.
C. Central necrosis with a neutrophilic infiltrate – Incorrect. A dense infiltrate of neutrophilic inflammation is typical for suppurative lymphadenitis.
D. Sheets and aggregates of atypical lymphoid cells – Incorrect. Sheets and aggregates of atypical lymphoid cells describe the histopathologic feature of lymphoma.
E. Atypical melanocytes with pagetoid spread – Incorrect. Atypical melanocytes with pagetoid spread are the histopathologic feature of primary melanoma.

**Question 3: Which of the following statements is true about this reaction secondary to pembrolizumab?**

A. SLRs are caused by direct cytotoxic effects of pembrolizumab.
B. Patients with SLRs require discontinuation of pembrolizumab due to poor prognostic outcomes.
C. The incidence of SLRs is higher with the use of immunotherapy for Merkel cell carcinoma compared to other cancers.
D. SLRs during pembrolizumab therapy have been correlated with improved overall survival.
E. Pembrolizumab is unique in that it is the only immunotherapy agent associated with an SLR.

**Answers:**

A. SLRs are caused by direct cytotoxic effects of pembrolizumab – Incorrect. Immune activation, not direct cytotoxicity, causes SLRs. Exact pathogenesis remains unknown, but likely involves enhanced T-cell activation due to PD-1 pathway inhibition.^2^ Lymphocyte sensitization to heavy metals in black tattoo ink may precipitate SLR with pulmonary involvement and trigger cutaneous granuloma formation due to the immune system’s perception of ink as foreign.
B. Patients with SLRs require discontinuation of pembrolizumab due to poor prognostic outcomes – Incorrect. Asymptomatic or mild cases do not require intervention, and immunotherapy can often be continued with careful monitoring. Treatment of SLRs, with systemic corticosteroids, should be considered when there are notable symptoms, organ dysfunction, or a negative impact on quality of life. However, providers should also consider the risk of diminishing immunotherapy effectiveness with such intervention.
C. The incidence of SLRs is higher with the use of immunotherapy for Merkel cell carcinoma compared to other cancers – Incorrect. SLRs are more common with melanoma immunotherapy, likely due to the high tumor immunogenicity and robust immune response it elicits.^3^
D. SLRs during pembrolizumab therapy have been correlated with improved overall survival – Correct. Multiple studies have demonstrated better outcomes in patients who develop SLRs during immunotherapy.^4^^,^^5^ One study of 434 patients undergoing immune-checkpoint inhibitor therapy found that patients who developed SLRs had significantly improved survival compared to patients who developed immune adverse events other than sarcoidosis reactions.^4^ However, SLRs can mimic disease progression or metastasis, clinically and radiographically, potentially prompting inappropriate treatment changes.
E. Pembrolizumab is unique in that it is the only immunotherapy agent associated with an SLR – Incorrect. SLRs have been associated with other agents: anti-CTLA-4 antibodies, anti-PD-L1 inhibitors, interferons, tumor necrosis factor-alpha inhibitors, and antiretroviral therapy.

## Conflicts of interest

None disclosed.
